# Ultrafast x-ray and electron scattering of free molecules: A comparative evaluation

**DOI:** 10.1063/4.0000010

**Published:** 2020-06-23

**Authors:** Lingyu Ma, Haiwang Yong, Joseph D. Geiser, Andrés Moreno Carrascosa, Nathan Goff, Peter M. Weber

**Affiliations:** Brown University, Department of Chemistry, Providence, Rhode Island 02912, USA

## Abstract

Resolving gas phase molecular motions with simultaneous spatial and temporal resolution is rapidly coming within the reach of x-ray Free Electron Lasers (XFELs) and Mega-electron-Volt (MeV) electron beams. These two methods enable scattering experiments that have yielded fascinating new results, and while both are important methods for determining transient molecular structures in photochemical reactions, it is important to understand their relative merits. In the present study, we evaluate the respective scattering cross sections of the two methods and simulate their ability to determine excited state molecular structures in light of currently existing XFEL and MeV source parameters. Using the example of optically excited N-methyl morpholine and simulating the scattering patterns with shot noise, we find that the currently achievable signals are superior with x-ray scattering for equal samples and on a per-shot basis and that x-ray scattering requires fewer detected signal counts for an equal fidelity structure determination. Importantly, within the independent atom model, excellent structure determinations can be achieved for scattering vectors only to about 5 Å^−1^, leaving larger scattering vector ranges for investigating vibrational motions and wavepackets. Electron scattering has a comparatively higher sensitivity toward hydrogen atoms, which may point to applications where electron scattering is inherently the preferred choice, provided that excellent signals can be achieved at large scattering angles that are currently difficult to access.

## INTRODUCTION

The determination of molecular structures using x-ray and electron scattering has found many applications in chemistry and related molecular sciences.^1–3^ The advent of technologies to create ultrafast pulses, first implemented on electron beams,^4–7^ has heralded an age where it is possible to measure molecular systems in transient excited states.^8–17^ More recently, x-ray Free-Electron Lasers (XFELs) and Mega-electron-Volt radio frequency (RF) electron guns have transformed the field, bringing the determination of transient molecular structures into a new era where interatomic distances are measured with sub-Ångström spatial resolution and femtosecond time resolution.^10,13,15,17–20^ This makes it possible to capture time-resolved molecular scattering signals revealing the structures of molecules during chemical reactions even in gas-phase small organic molecules.^21–24^ The high brightness of XFELs and MeV RF guns makes it possible to study the photochemistry in low-density gas phase vapors, where the molecular motions can be isolated without interference of nearby molecules as reaction dynamics proceed. The gas-phase molecular dynamics are important since they provide a direct comparison between experimental measurements^25^ with detailed theoretical calculations^26^ and provide a crucial source of reference data.^27,28^ Important applications of these high-brightness, short pulse duration beams include the determination of molecular structures in excited states^29–31^ and the observation of ultrafast dynamics of photoexcited chemical reactions.^21,22,25,32^

**FIG. 1.**
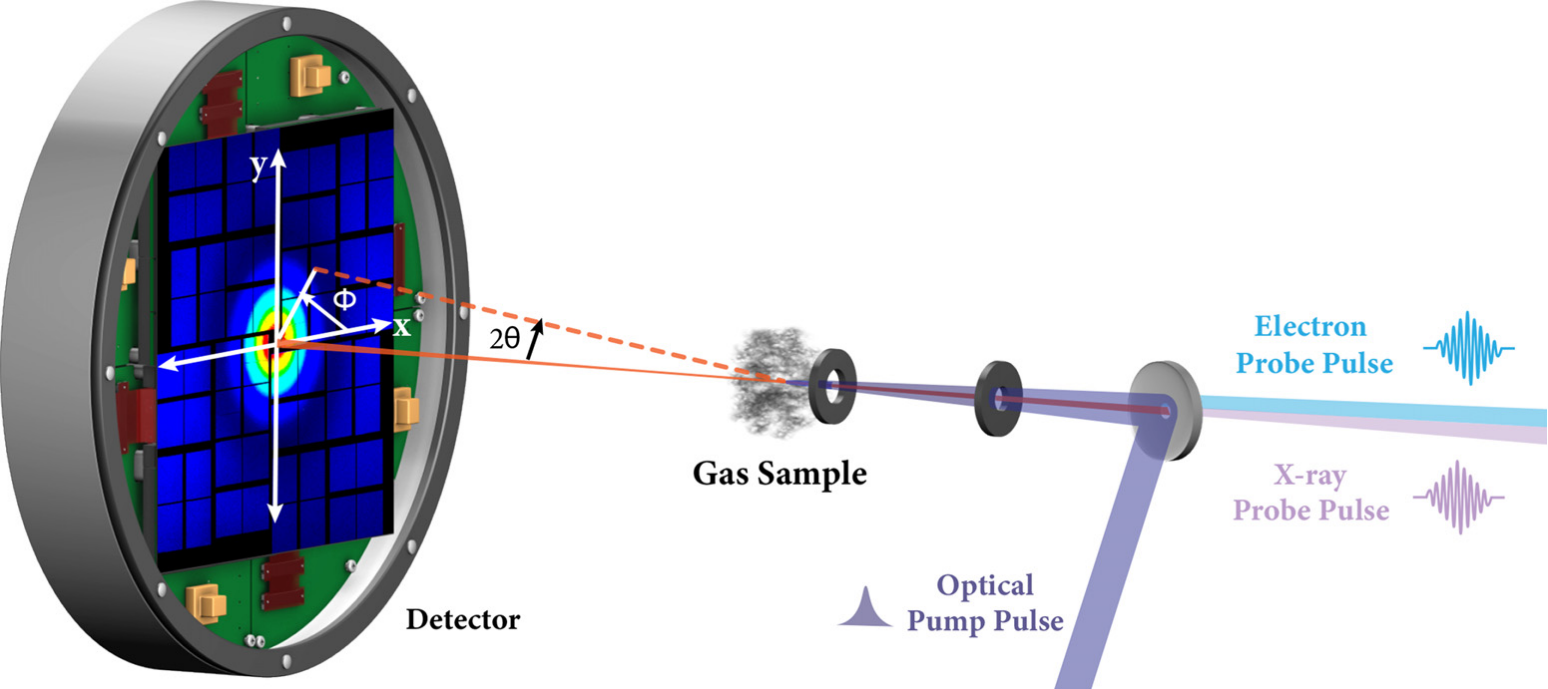
A typical pump-probe experimental setup for ultrafast x-ray and electron scattering. An optical pump pulse initiates a photochemical reaction, and time-dependent x-ray or electron scattering patterns are created by the time-delayed interaction with the probe pulse. The scattering signals are recorded as a function of the scattering angle 2θ and the azimuthal angle Φ.

Scattering experiments, i.e., ultrafast x-ray scattering and ultrafast electron diffraction (UED), could, in principle, offer direct access to complete molecular structures. To measure their time evolution, a pump-probe scheme is the most commonly used experimental technique. A pump laser pulse initiates the photochemical reaction, and after a specific delay time, an x-ray or electron probe pulse casts a scattering pattern that maps the transient molecular structure^25,33–35^ (Fig. 1). Determining dynamic motions of the molecule requires that the optical laser pump pulse induces a deterministic photochemical dynamics. The time resolution is limited by the duration of the laser and the x-ray or electron pulses and the timing jitter between them. By collaging the individual diffraction patterns in temporal order, one can decode the transient structures and produce a “molecular movie” of the molecular motions.^29^

X-ray scattering by a free charged particle follows classical Thomson scattering, which describes the elastic scattering of electromagnetic radiation. X-ray scattering by molecular systems is therefore dominated by their interaction with the electrons in the molecule.^36–38^ On the other hand, electron scattering follows Rutherford scattering and depends on the electrostatic Coulomb interaction of the traveling electrons with all charged particles in the molecule. Although x-ray scattering and electron scattering are equally applied toward structure determination, various capabilities and advantages arising from their different physical interactions have been discussed previously.^37,39,40^

In this article, we aim to explore the relative capabilities of x-ray and electron scattering toward the determination of excited state structures. We first compare the relative scattering cross sections by evaluating the probability for a single molecule to scatter an x-ray or an electron. We use the independent atom model (IAM), which is sufficiently accurate for the present comparison in describing pump-probe scattering patterns, even though it partially ignores important contributions arising from the changing electron densities, i.e., the density between chemical bonds, upon optical excitation.^41–44^ As a model system, we chose N-methyl morpholine (NMM), where Stankus and Yong *et al.*^29^ measured the coherent structural dynamics upon optical excitation. To mimic the experiments, we simulate gas-phase x-ray and electron total scattering patterns containing different magnitudes of shot noise while neglecting other sources of noise that may be specific to individual experiments.

Our comparison is grounded in current technologies. For the x-ray beam, we assume parameters from the Linac Coherent Light Source (LCLS),^21,23,24^ while parameters for electron scattering are selected from “table-top” time-resolved electron scattering instruments^8,9,17,45^ and for the relativistic UED system at the SLAC National Accelerator Laboratory.^20,22,46–48^ Relativistic electron beams, with energies in the MeV range, are particularly useful for pulsed experiments as they minimize broadening of the temporal resolution on account of space-charge interactions between electrons within a pulse.^49–51^ Relevant beam parameters are listed in Table I. Following tradition in the respective literature, we denote the momentum transfer either as ***q*** or ***s*** for x-ray and electron scattering, respectively. This makes transparent to which method a formula or figure relates.

**TABLE I.** X-ray and electron pulse parameters used for the comparative simulations.

	Energy	Wavelength	Photons or electrons per pulse, $P_{0}$	Pulse repetition rate	Pulse duration
X-ray^a^	8.3 keV	1.494 Å	$10^{12}$ photons/pulse	120 Hz	30 fs
MeV-UED^b^	3.7 MeV	0.003Å	$3.7\times 10^{4}$ e^−^/pulse	120 Hz	230 fs
keV-UED^c^	30 keV	0.067 Å	$2.5\times 10^{3}$ e^−^/pulse	50 000 Hz	> 1 ps

^a^ Data from Ref. 24. To allow a comparison up to scattering vectors of 20 Å^−1^, the simulations use a photon energy of 39.5 keV.

^b^ Data from Ref. 46, with a charge of 6 fC delivered to the sample. According to the SLAC National Accelerator Laboratory website, the facility is now offering repetition rates up to 360 Hz pulse and pulse durations shorter than 150 fs, dependent on the beam charge.^52^ In order to shorten the pulse duration of relativistic electron pulses, many important advances have been made,^13,15,53–58^ especially including radio frequency (RF)-based velocity compression techniques.^54–58^

^c^ Data from Ref. 8. Other keV electron beam sources with RF compression have reported sub-picosecond pulses, where the temporal resolution was limited only by RF electronic synchronization noise.^17,45,54,59,60^ The synchronization noise in keV-UED-RF has been recently solved.^61^ Furthermore, compression of keV electron pulses with terahertz radiation offers new opportunities for femtosecond gas-phase scattering.^62^ Compact electron sources capable of providing ∼300 fs pulses with $10^{4}e^{-}$/pulse may be suitable for some investigations.^63^

We find that although x-ray scattering has a much smaller cross section than electron scattering, the dramatically larger number of photons per pulse makes the probability of scattering an x-ray out of a pulse 300–1000 times larger than that of scattering an electron from a current-technology relativistic electron pulse. Applying the structure determination method introduced by Stankus and Yong *et al.*,^29^ we investigate the effects of shot noise and the range of momentum transfer vectors on the accuracy of the structure determination with x-rays and electrons, respectively. It is seen that with current instrumentation, for equal samples and on a per-shot basis, the fidelity for structure determination is superior with x-rays. Importantly, expanding the range of scattering vectors while keeping the same number of scattered particles introduces noise to the detriment of structure determination. Indeed, a sufficient structure determination for gas-phase small organic molecules free of heavy atoms can be achieved already with a range of scattering vectors from 0.5 to 5 Å^−1^. The IAM model enables us to isolate contributions from different interatomic distances to the pump-probe scattering patterns. This analysis suggests that both x-ray and electron scattering might be able to determine H atom positions. The more favorable form factors give electron scattering the edge, however, only if very high quality signals can be measured.

## THEORY AND COMPUTATIONAL METHODS

### Thomson and Rutherford Scattering

Classical x-ray scattering by a free, charged particle is described by elastic Thomson scattering of electromagnetic radiation. For unpolarized x-ray beams, the intensity of scattering by a single free electron is^36^

$$I_{\mathrm{scatt}}^{Th}\left(2\theta \right)=I_{0}\frac{e^{4}}{{m_{e}}^{2}c^{4}R^{2}}\left(\frac{1+{\text{cos}}^{2}\left(2\theta \right)}{2}\right),$$ (1)

where $I_{0}$ and $I_{\mathrm{scatt}}^{Th}$ are the incoming and scattered intensities (W/${\mathrm{cm}}^{2}$), *R* is the distance to the detector, $\frac{{d\sigma }_{Th}}{d\Omega }=\frac{e^{4}}{m^{2}c^{4}}=7.94\times 10^{-26}\;{\mathrm{cm}}^{2}$ is the differential Thomson cross section for a free electron, $\frac{1+{\text{cos}}^{2}(2\theta )}{2}$ is the polarization factor for an unpolarized incident beam, $m_{e}$ and *e* are the mass and charge of an electron, and *c* is the velocity of light. It is worth noting that the polarization factor also applies to linearly polarized x-rays when azimuthally averaging scattering signals from an isotropic ensemble of molecules. The scattering angle 2θ, Fig. 1, is related to the magnitude of the momentum transfer vector by $q=\left|k-k_{0}\right|=2k_{0}\;\text{sin}\;\theta$, where $k_{0}$ is the incident wave vector and $\left|k_{0}\right|=\frac{2\pi }{\lambda }=\frac{2\pi E}{hc}$ and k is the scattered wave vector. The total scattering cross section is found by integrating over the Ewald sphere, $\sigma_{Th,\mathrm{tot}}=\frac{8\pi }{3}\frac{{d\sigma }_{Th}}{d\Omega }$.

For our comparative analysis, we are interested in the number of scattered photons, $P_{\mathrm{scatt},X}$, for a given number of photons in the incoming x-ray pulse, $P_{0,X}$. The photon numbers relate to the intensities by

$$I_{0}=P_{0,X}\frac{h\cdot \nu \cdot R_{r}}{A_{0}}\;\text{and}\;I_{\mathrm{scatt}}^{Th}=P_{\mathrm{scatt},X}\frac{h\cdot \nu \cdot R_{r}}{A},$$

where $R_{r}$ is the repetition rate of the XFEL, h·ν is the photon energy (approximated to be equal for the incoming and outgoing beams), and $A_{0}$ and A are the areas of the incoming x-ray beam and the unit area on the detector, respectively. To calculate the probability of photons scattered into a resolution element Δq of the scattering vector, integrated over all azimuthal angles, we integrate Eq. (1) over the circular area element between q and q+Δq in the momentum transfer space (Fig. S1). The probability of photons scattered into a resolution element Δq at the momentum transfer vector q is then given by

$$\frac{P_{\mathrm{scatt},X}(q,\;q+\Delta q)}{P_{0,X}}=\frac{{d\sigma }_{Th}}{d\Omega }\cdot \frac{1}{A_{0}\cdot 2k_{0}^{2}}\cdot \left(1+{\text{cos}}^{2}\left(2a\mathrm{rcsin}\frac{q}{2k_{0}}\right)\right)\cdot 2\pi q\cdot \Delta q.$$ (2)

The scattering signal scales as the area of the resolution element 2πqΔq and the ${\text{cos}}^{2}(2\theta )$ dependence on the scattering angle. The $\frac{1}{2k_{0}^{2}}$ term suggests that higher photon energies give a smaller scattering probability into a momentum transfer resolution element.

Electron diffraction is determined by Rutherford scattering, which arises from the Coulomb interactions between charged particles. Using the first Born approximation, the Mott-Born cross section describes the differential scattering cross section of a relativistic electron by a point charge Z,^49–51,64^

$$I_{\mathrm{scatt}}^{{Ru}^{\prime }}\left(2\theta \right)=I_{0}\cdot \frac{1}{R^{2}}\cdot {\left(\frac{2m_{e}{Ze}^{2}}{\hbar^{2}}\right)}^{2}\cdot \frac{1}{s^{4}}\cdot \left(\frac{1-\beta^{2}\;{\text{sin}}^{2}\theta }{1-\beta^{2}}\right),$$ (3)

where *Z* is the charge of the target particle, *m_e_* is the mass of the scattered electron, $s=2k_{0}\;\text{sin}\;\theta$ is the magnitude of the scattering vector (named q in x-ray scattering), and β=vc is the electron velocity in units of the speed of light. In the Mott-Born cross section, $I_{Ru}^{\prime }d\omega$ refers to the number of scattered electrons into the solid angle dω per unit time. For non-relativistic electrons, $\left(\frac{1-\beta^{2}\;{\text{sin}}^{2}\theta }{1-\beta^{2}}\right)\approx 1$ so that the scattered intensity approaches the expression reported by Mott and Massey,^65^

$$I_{\mathrm{scatt}}^{{Ru}^{\prime }}\left(2\theta \right)\approx I_{0}\cdot \frac{1}{R^{2}}\cdot {\left(\frac{2m_{e}Z\cdot e^{2}}{\hbar^{2}}\right)}^{2}\cdot \frac{1}{s^{4}}.$$

To calculate the probability of electrons scattered into a resolution element Δs, we convert Eq. (3) into a form that gives the electron scattering into the circular area between s and s+Δs in the momentum transfer space (Fig. S1) to get

$$\frac{P_{\mathrm{scatt},e}(s,s+\Delta s)}{P_{0,e}}=\frac{{d\sigma }_{Ru}}{d\Omega }\cdot \frac{1}{A_{0}\cdot 4k_{0}^{2}}\cdot \frac{1}{\sqrt{1-{\left(\frac{s}{2k_{0}}\right)}^{2}}}\cdot \left(\frac{1-\beta^{2}\;{\text{sin}}^{2}\theta }{1-\beta^{2}}\right)\cdot 2\pi s\cdot \Delta s,$$ (4)

where $\frac{{d\sigma }_{Ru}}{d\Omega }={\left(\frac{Z\cdot Z^{\prime }\cdot e^{2}}{2m_{e}v^{2}}\right)}^{2}\cdot {\text{cosec}}^{4}\theta ={\left(\frac{2Z\cdot Z^{\prime }\cdot m_{e}e^{2}}{\hbar^{2}s^{2}}\right)}^{2}=Z^{2}\cdot {\left(\frac{2m_{e}e^{2}}{\hbar^{2}}\right)}^{2}\cdot \frac{1}{s^{4}}$ when the incident particle is an electron. The Rutherford scattering cross section, $C_{Ru}={\left(\frac{2m_{e}e^{2}}{\hbar^{2}}\right)}^{2}=1.428\times 10^{-15}{\mathrm{cm}}^{2}\cdot {Å}^{4},$ is much larger than the Thomson cross section, but Eqs. (2) and (4) are very similar. The interaction between charged particles causes the $\frac{1}{s^{4}}$ term in the Rutherford scattering, which diverges at s = 0, making it impossible to calculate the total scattering cross section for a single charge.

### Scattering off atoms and molecules

The electrons in atoms and molecules are not point charges, and their extended shape needs to be taken into account. For x-ray scattering, the elastic atomic form factor,

$$f_{x}\left(q\right)=F\left[\rho \left(r\right)\right]=\int \rho \left(r\right)e^{iq\cdot r}dr,$$ (5)

is the Fourier transform of the electron density $\rho \left(r\right)$ of the atom, which can be obtained from electronic structure calculations. Convenient tabulations exist for x-ray form factors for all the atomic elements.^66^

Electrons scatter off the total charge density of a target atom, and therefore, the atomic form factor for electrons is the Fourier transform of the atomic potential $V\left(r\right),$^50,67^

$$f_{e}\left(s\right)=\frac{2m_{e}}{\hbar^{2}}F\left[V\left(r\right)\right]=\frac{2m_{e}}{\hbar^{2}}\int V(r)e^{is\cdot r}dr.$$ (6)

**FIG. 2.**
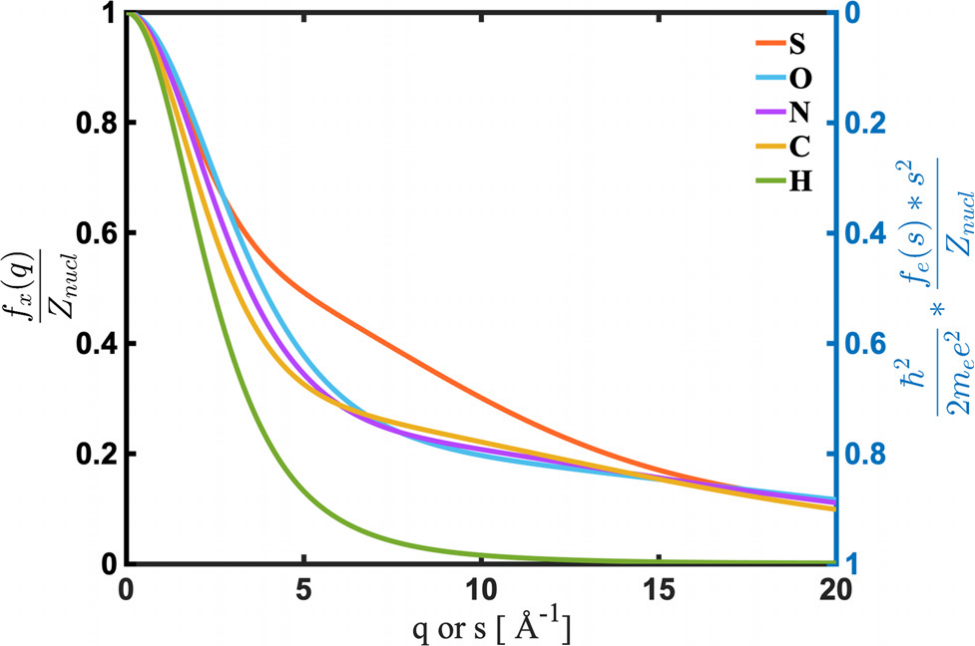
The atomic form factors of sulfur (S, red), oxygen (O, light blue), nitrogen (N, purple), carbon (C, orange), and hydrogen (H, blue) atoms, for x-ray scattering (left axis) and electron scattering (right axis), respectively. The form factors are scaled by the atomic number $Z_{\mathrm{nucl}}$, and the electron form factors are in addition scaled by the Rutherford cross section prefactors.

Electron form factors can be computed from the Fourier transform of the atomic potentials^49,68–70^ or found as approximate analytical expressions.^71,72^ It is most convenient to use the Mott–Bethe formula, which relates electron scattering form factors to x-ray atomic scattering factors,^49^

$$f_{e}\left(s\right)=\frac{2m_{e}e^{2}}{\hbar^{2}}\cdot \frac{Z_{\mathrm{nucl}}-f_{x}\left(s\right)}{s^{2}},$$ (7)

where $Z_{\mathrm{nucl}}$ is the atomic number of the atom. Equation (7) suggests that outside of the constants and the $\frac{1}{s^{2}}$ dependence of the Rutherford cross section, electron scattering and x-ray scattering have fundamentally the same information content in that both depend on the x-ray form factor. Yet, they are also complementary: while the x-ray form factors decrease with the increasing scattering angle, the electron form factors increase in an inverse manner (Fig. 2). The increasing electron form factors partially compensate for the dramatic loss of electron scattering signals arising from the rapidly decaying $\frac{1}{s^{4}}$ term of Rutherford scattering.

Elastic scattering off molecules follows Eqs. (5) and (6). However, rather than calculating molecular densities or potentials for each molecule, practitioners in the field often invoke the independent atom model (IAM) approximation. The IAM treats the molecular electron density as a sum of spherical atomic densities, each centered at the positions of the nuclei. The molecular form factor $f_{\mathrm{IAM}}(q)$ is given by

$$f_{\mathrm{IAM}}\left(q\right)=\sum_{\alpha =1}^{N_{at}}f_{\alpha }\left(q\right)e^{ir_{\alpha \;\cdot }\;q},$$ (8)

where $f_{\alpha }\left(q\right)$ are the atomic form factors for x-ray and electron scattering, respectively.^73^ The summation extends over all $N_{at}$ atoms in the molecule with positions $r_{\alpha }$.

The form factors modify the amplitude of the scattering signal so that formulas (1) and (3) are modulated by the square of the molecular form factor (8). This leads to terms containing only the atomic scattering factors (often called “atomic scattering”) and cross terms that depend on the interatomic distances $r_{\alpha \beta }={|r}_{\alpha }-r_{\beta }|$ (called molecular scattering). Averaging over the random orientations of gas phase molecules following Debye^74^ results in

$$F_{M,x}\left(q\right)=\sum_{a=1}^{N_{at}}\left({\left|f_{\alpha }(q)\right|}^{2}+S_{\alpha }\right)+\sum_{\alpha \neq \beta }^{N_{at}}f_{\alpha }(q)f_{\beta }(q)\frac{\text{sin}(qr_{\alpha \beta })}{qr_{\alpha \beta }},$$ (9)

$$F_{M,e}\left(s\right)=\sum_{\alpha =1}^{\mathrm{Nat}}\left({\left|Z_{\mathrm{nucl}}^{\alpha }-f_{\alpha }\left(s\right)\right|}^{2}+S_{\alpha }\right)+\sum_{\alpha \neq \beta }^{N_{at}}(Z_{\mathrm{nucl}}^{\alpha }-f_{\alpha }\left(s\right))(Z_{\mathrm{nucl}}^{\beta }-f_{\beta }\left(s\right))\frac{\text{sin}({sr}_{\alpha \beta })}{sr_{\alpha \beta }},$$ (10)

for the rotationally averaged, isotropic total x-ray and electron scattering signals, respectively. Included in Eqs. (9) and (10) are the inelastic scattering terms, $S_{\alpha }$, which, in both x-ray and electron scattering, are caused by Compton scattering. The inelastic terms coming from Compton corrections are not dependent on the molecular geometry. While the inelastic terms mask the complexity of the inelastic scattering processes,^75^ they are conveniently obtained from the tabulated incoherent scattering functions of the elements.^76,77^

The IAM neglects completely the formation of chemical bonds, the polarization of atoms in a molecule, and the electronic excitations. Nevertheless, it approximates observed scattering signals reasonably well. To be sure, the fitting of high-quality scattering data requires an accurate description of electron densities.^29,30,78^ In particular, the change in electron density distributions must be understood to model the changes in the electronic structure upon optical excitation.^29^ Nevertheless, for the comparative evaluation of ultrafast x-ray and electron scattering signals, the IAM model provides a reasonable approximation of the scattering signals.

### Observed x-ray and electron scattering signals

From the discussion, it is clear that observed scattering signals depend on both the fundamental scattering cross sections for x-rays and electrons, on the composition and structure of the investigated molecules, and on the range of scattering angles subtended by the detector. We use Eqs. (2) and (9) to express the probability of scattering x-ray photons, i.e., the ratio of scattered to incoming photon numbers per pulse, from N molecules into a range of scattering vectors between $q_{1}$ and $q_{2},$

$$\frac{P_{\mathrm{scatt},X}(q_{1},\;q_{2})}{P_{0,X}}=\frac{{d\sigma }_{Th}}{d\Omega }\cdot \frac{\pi N}{A_{0}k_{0}^{2}}\int_{q_{1}}^{q_{2}}F_{M,x}\left(q\right)\cdot \left(1+{\text{cos}}^{2}\left(2a\mathrm{rcsin}\frac{q}{2k_{0}}\right)\right)\cdot q\cdot dq.$$ (11)

The number of molecules can be estimated for the given experimental conditions and the geometry of the scattering cell, in particular the path length. The number of incoming x-ray photons is also determined in an experiment so that approximate absolute numbers of scattered photons can be calculated.

For electron scattering, the ratio of scattered to incoming electron numbers per pulse from N molecules into a range of scattering vectors between $s_{1}$ and $s_{2}$ is

$$\frac{P_{\mathrm{scatt},e}(s_{1},\;s_{2})}{P_{0,e}}=C_{Ru}\cdot \frac{\pi N}{{2A}_{0}k_{0}^{2}}\cdot \int_{s_{1}}^{s_{2}}F_{M,e}\left(s\right)\cdot \frac{1}{s^{3}}\cdot \frac{1}{\sqrt{1-{\left(\frac{s}{2k_{0}}\right)}^{2}}}\cdot \left(\frac{1-\beta^{2}{\left(\frac{s}{{2k}_{0}}\right)}^{2})}{1-\beta^{2}}\right)\cdot ds,$$ (12)

where again all parameters can be determined from the experimental conditions or calculated for specific molecules. Equations (11) and (12) give the total scattering signals. The measured signals are usually smaller on account of inefficiencies of the detectors used.

Even though XFEL and UED experiments use different detectors to capture time-dependent scattering patterns, e.g., the Cornell-SLAC Pixel Array Detector^79^ for x-rays or a P43 phosphor screen imaged on a cooled Electron Multiplying Charge Coupled Device^52^ for electrons, both can image scattered particles on a per-shot basis with high efficiency and exhibit excellent spatial resolution over a large solid angle.^80,81^ The detector technologies develop rapidly, and more detailed information can be found in the literature.^20,82–85^

### Noise in the scattering signals

There are many sources of noise in pump-probe diffraction experiments, including, in particular, the intensity fluctuations of the pulse stream of the XFEL and the UED. While many details depend on the experimental implementations, experience shows that many such noise sources can be eliminated or accounted for. This leaves as the ultimate noise of both x-ray and electron scattering experiments the statistical counting noise, which is the only noise considered here.

In a scattering experiment, the number of particles $P_{\mathrm{scatt}}$ scattered onto a certain detector area obeys the Poisson distribution. The noise of the measurement, i.e., the standard deviation of the total counts, is the square root of the observed number of scattered particles. Thus, the signal to noise ratio of a scattering experiment can be expressed by

$$\mathrm{SNR}=\frac{\mathrm{Signal}}{\mathrm{Noise}}=\frac{P_{\mathrm{scatt}}}{\sqrt{P_{\mathrm{scatt}}}}=\sqrt{P_{sc\mathrm{att}}}.$$ (13)

In pump-probe experiments, we consider the changes in the scattering signals upon optical excitation, i.e., the fractional difference between the signal with the optical pump laser on, $P_{on}\left(q\right)$, and the laser off, $P_{\mathrm{off}}\left(q\right),$

$$\Delta S\left(q\right)=\frac{P_{on}\left(q\right)-P_{\mathrm{off}}\left(q\right)}{P_{\mathrm{off}}\left(q\right)}.$$ (14)

Assuming that the laser-off signal can be measured with negligible statistical counting noise as is often the case, the noise can be expressed as

$$\Delta \left(\Delta S(q)\right)=\frac{\sqrt{P_{on}(q)}}{P_{\mathrm{off}}(q)}.$$ (15)

The SNR is then

$$\mathrm{SNR}\left(q\right)=\frac{P_{on}\left(q\right)-P_{\mathrm{off}}(q)}{\sqrt{P_{on}(q)}}.$$ (16)

To simulate the noise, we assume that each photon or electron is scattered off photo-excited molecules with a scattering probability distribution calculated from Eqs. (9) or (10). The simulations assume low intensities so that one photon/electron scattering processes dominate. The scattering vectors are analyzed with a bin size of 0.1 Å^−1^ for both x-ray and electron scattering. We iterate this scattering process $N_{\mathrm{total}}$ times with each scattering process generated randomly, where $N_{\mathrm{total}}$ is the total number of scattered photons or electrons. We sum up the particle counts in each bin to obtain the statistical scattering intensity that contains the Poisson-distribution shot noise.

### Probability of optical excitation

The exposure of molecules to intense laser radiation can readily induce multi-photon processes in addition to single-photon excitation. It is nearly impossible to prepare a reproducible, well-defined starting state for chemical dynamics if there is a significant portion of multi-photon excitation on account of excessive optical pulse intensity. Consequently, the probability of excitation, γ, should be kept quite low, usually less than 0.10. This low probability of excitation increases the demand for high quality signals but is essential to achieve accurate determinations of transient molecular structures.

The fractional difference signal including an excitation probability γ is expressed by

$$\Delta S_{\gamma }(q)=\gamma \cdot \frac{P_{on}\left(q\right)-P_{\mathrm{off}}\left(q\right)}{P_{\mathrm{off}}\left(q\right)}\;.$$ (17)

While the pump-probe signal is reduced by the excitation probability, the shot noise remains the same. Thus, the SNR with excitation γ is

$${\mathrm{SNR}}_{\gamma }\left(q\right)=\gamma \cdot \frac{P_{on}\left(q\right)-P_{\mathrm{off}}\left(q\right)}{\sqrt{P_{on}\left(q\right)}}.$$ (18)

Because the SNR scales roughly with the square root of the signals, in order to achieve the same SNR even with excitation probability γ, the signal needs to be scaled up by a factor of $1/\gamma^{2}$. In our comparative analysis, we show results for 100% excitation, but we emphasize that for a comparison to experimental data, this factor needs to be taken into account.

### Structure determination and method assessment

For our comparative analysis, we adopt the structure determination methodology introduced by Stankus and Yong *et al.*^29^ (see details in the supplementary material). In order to determine the effects of shot noise and the range of the observed momentum transfers for x-ray and electron scattering on the accuracy of the structure determination, we simulate the signals based on Eqs. (11) and (12) for a specific excited state structure. To include the effect of noise, a synthetic noise is overlaid over the noise-free, theoretical patterns as discussed above.

The accuracy of this structure determination method is limited by the number of unique structures in the pool, with the value of the limit dependent slightly on the weighing of the data points and the functional dependence on the scattering angle. Empirically, we find that the limiting values of the average percent deviations are 0.6% for x-ray and 0.8% for electron scattering. In the data reported below (in particular Fig. 5), we subtract out those limiting values.

This method is limited in extracting the evolving nuclear wavepacket and is used solely to extract excited state structures. It relies on the approximation of the excited state structure by a single classical molecular structure, though it can be extended to the case where the wavepacket bifurcates into multiple channels if an adequate number of geometries from both channels are included in the pool of structures. We will next compare x-ray and electron scattering on their ability to select excited state structures within this framework.

## COMPARATIVE EVALUATION OF A MODEL SYSTEM

### Total scattering signals

While Eqs. (11) and (12) are quite general, the amount of scattering depends on the atomic composition and the structure of the molecule. In our comparative analysis, we investigate a representative heterocyclic organic molecule, N-methyl morpholine, C_5_H_11_NO, where Stankus and Yong *et al.* have extensively studied the photo-induced dynamics.^29^ For the present study, we employed their structure pools to perform our structure determination, and we model the signals based on a hypothetical excited state structure that is taken to be the structure of the ionic ground state.^29^ Since our simulations use the IAM model, the change in the electron density upon electronic excitation is neglected, even though it can be an observable effect.^86^

**FIG. 3.**
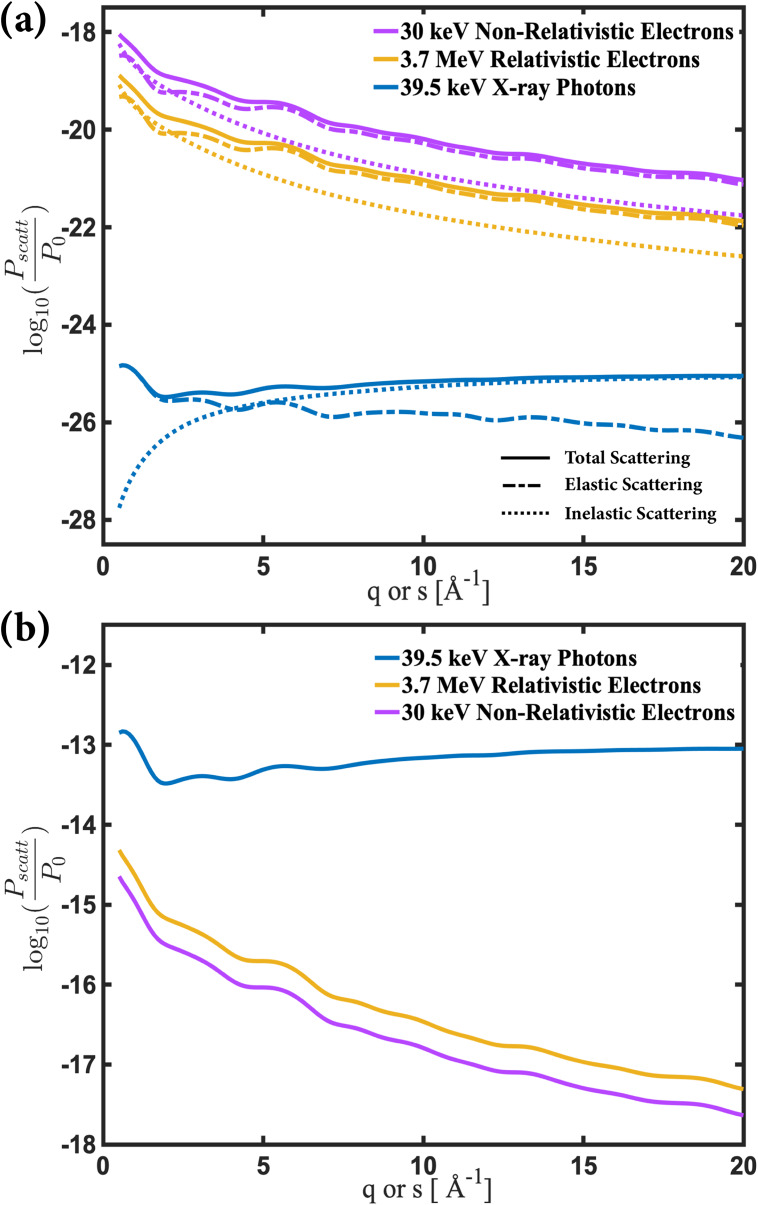
(a) The total (solid line), elastic (dashed line), and inelastic (dotted line) differential scattering probabilities, integrated over all azimuthal angles for a 1 cm^2^ beam area, 1 target NMM molecule, and 1 probe particle (x-ray photon or electron). (b) The total scattered probe particle counts by 1 target molecule for a typical x-ray pulse or electron pulse, for 39.5 keV x-ray photons, 3.7 MeV relativistic electrons, and 30 keV non-relativistic electrons. The probabilities refer to scattering into a resolution element with $\Delta q=0.1\;{Å}^{-1}$.

Based on Eqs. (11) and (12) for x-rays and electrons, respectively, we evaluate the number of scattered photons or electrons in a scattering experiment. We consider first the hypothetical case where one incoming probe particle (photon or electron) encounters one target molecule (N=1). The scattering probability is dependent on the area of the beam focus ($A_{0}$), which we take to be 1 cm^2^, as well as the momentum ($k_{0}=\frac{2\pi }{\lambda }=\frac{2\pi E}{hc}$) of the incoming probe particle. The dependence of this scattering signal on the absolute value of the scattering vector, Fig. 3(a), is evaluated for energies that match experimental implementations, Table I, namely, 39.5 keV for the x-rays and 3.7 MeV and 30 keV for relativistic and non-relativistic electrons, respectively.

Because the Thomson cross section is much smaller than the Rutherford cross section, electrons have a much higher scattering probability than photons for the same number of incident particles in the beam. The total x-ray scattering signal flattens out at larger *q* because while the elastic scattering continues to drop, the inelastic contribution increases with *q*. Although the differential cross section for relativistic electrons is about 100× larger than for non-relativistic electrons, the $\frac{1}{k_{0}^{2}}$ dependence in formula (12) reduces the scattering probability for relativistic electrons, making it smaller than that of non-relativistic electrons by about an order of magnitude.

The total scattering probability, i.e., the elastic plus inelastic scattering integrated over the observed range of scattering vectors, is also dependent on the molecular composition. Table II reports the total scattering probabilities for the three representative beam parameters and our chosen NMM target molecule.

**TABLE II.** The integrated probability, PscattP0, for one probe particle scattered off one target NMM molecule within momentum transfers from 0.5 to 20 Å^−1^ and the total count of scattered particles, $P_{\mathrm{scatt}}$, using $P_{0}$ values from Table I.

	39.5 keV x-rays	30 keV electrons	3.7 MeV electrons
Total scattering probability, PscattP0	1.36 × 10^–23^	9.01 × 10^–18^	1.30 × 10^–18^
Scattered particle count, $P_{\mathrm{scatt}}$	1.36 × 10^–11^	2.25 × 10^–14^	4.81 × 10^–14^

In actual experiments, the beam parameters obtained with current instrumentation are very different. The LCLS x-ray free electron laser produces ultrashort x-ray pulses containing about 10^12^ photons each.^24^ The number of electrons in an electron pulse is constrained by space-charge interactions. In the non-relativistic experiment with picosecond duration pulses, there were about $2.5\times 10^{3}$ electrons/pulse.^8^ Shorter duration pulses are limited to fewer electrons per pulse, although recompression techniques may make it possible to sustain larger electron counts. Relativistic electron beams are less affected by space-charge interactions, making femtosecond duration pulses possible while achieving $3.7\times 10^{4}$ electrons/pulse.^46^ Figure 3(b) expresses the scattering probabilities for one target molecule and for those beam parameters. It is seen that even though the Thomson scattering cross section is up to 10^5^ times smaller than the relativistic Rutherford scattering, since there are >10^7^ times more photons in an x-ray pulse than there are electrons in an electron pulse, the total scattering for an x-ray pulse is 300–1000 times larger than that for a relativistic electron pulse. Thus, with current technology, the x-ray scattering produces a much higher signal on a per-pulse and per-target molecule basis.

#### Pump-probe signals

We use the IAM to calculate the scattering signals and counting noise for the excitation of NMM with ground state and excited electronic state structures as reported by Stankus and Yong *et al.*^29^ The simulations cover the range of scattering vectors from 0.5 ${Å}^{-1}$ up to 20 ${Å}^{-1}$. We assume 100% excitation but emphasize that to get meaningful results, much smaller excitation probabilities should be used. We calculate the noise for the cases of 1 million and 30 million total scattered photons or electrons, while noting that based on the results of Fig. 3 and Table II, it takes very different data acquisition times to achieve those signals.

**FIG. 4.**
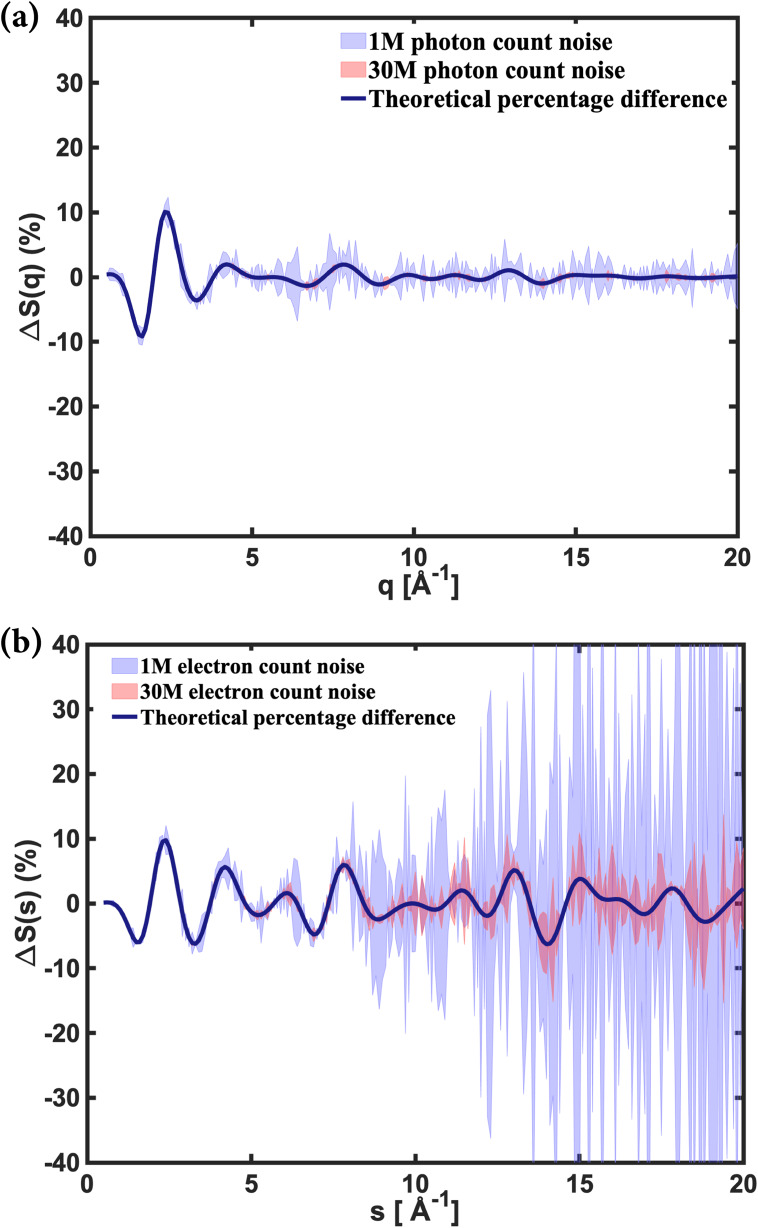
The fractional difference scattering signals calculated for (a) x-ray and (b) electron total scattering for the optical excitation of NMM, solid lines, assuming 100% excitation. The noise is simulated for 10^6^ (purple) and 3 × 10^7^ (pink) scattered photons or electrons, respectively.

Figures 4(a) and 4(b) show the fractional difference in scattering signals for x-ray and electron scattering, respectively, with noise as given by Eq. (15). It is obvious that larger signals reduce the noise as expected.

For x-ray scattering, we find that the shot noise approaches a constant magnitude for *q* larger than about 5 Å^−1^, which is traced to the rise of the inelastic scattering signal. While for 1M detected photons, the noise is significant, the noise in the 30M simulation is nearly imperceptible. In the actual experiment of Stankus and Yong *et al.*,^29^ about 5000 shots were acquired for each delay time point. With each shot yielding about 50 000 counts, this implies that 2.5 × 10^8^ counts were detected. In that experiment, the excitation was kept to 5.7%. Scaling the total counts down by the excitation probability would imply that the noise in the experiment was approximately equivalent to the 1M count simulation. The noise in the reported experiment is consistent with our calculation. We note that to acquire the 5000 shots takes less than a minute.

For relativistic electron scattering, Fig. 4(b), the noise increases dramatically with *s* to the point where it obscures the information carried by the scattering signals. This is, of course, the result of the rapidly decaying $\frac{1}{s^{4}}$ dependence in electron scattering. Up to about 5 Å^−1^, this decay is countered by the increasing form factors, Fig. 2, but for larger scattering vectors, the rapid decay prevails. For the 1M simulation, the noise overshadows the scattering signals beyond 8 Å^−1^. Even in the 30M simulation, the scattering signals are compromised for *s* larger than 10 Å^−1^. These results seem broadly in line with the published experimental results,^22^ but we note that to achieve the same noise level and assuming an equal optical excitation, about 10^5^ times more shots are needed for MeV-UED compared to x-ray scattering (Table II).

#### Structure determination

In order to investigate how noise and the range of momentum transfers affect the accuracy of structure determination, we take the simulated noisy difference patterns as the input for the structure analysis method. The phase space covered by the pool of possible molecular structures has been discussed in Ref. 29. Since these patterns were generated using a specific excited state structure, the structure obtained from the analysis can be directly evaluated for its accuracy, with smaller average percent deviations as defined in equation (S1), implying a higher accuracy of the structure.

**FIG. 5.**
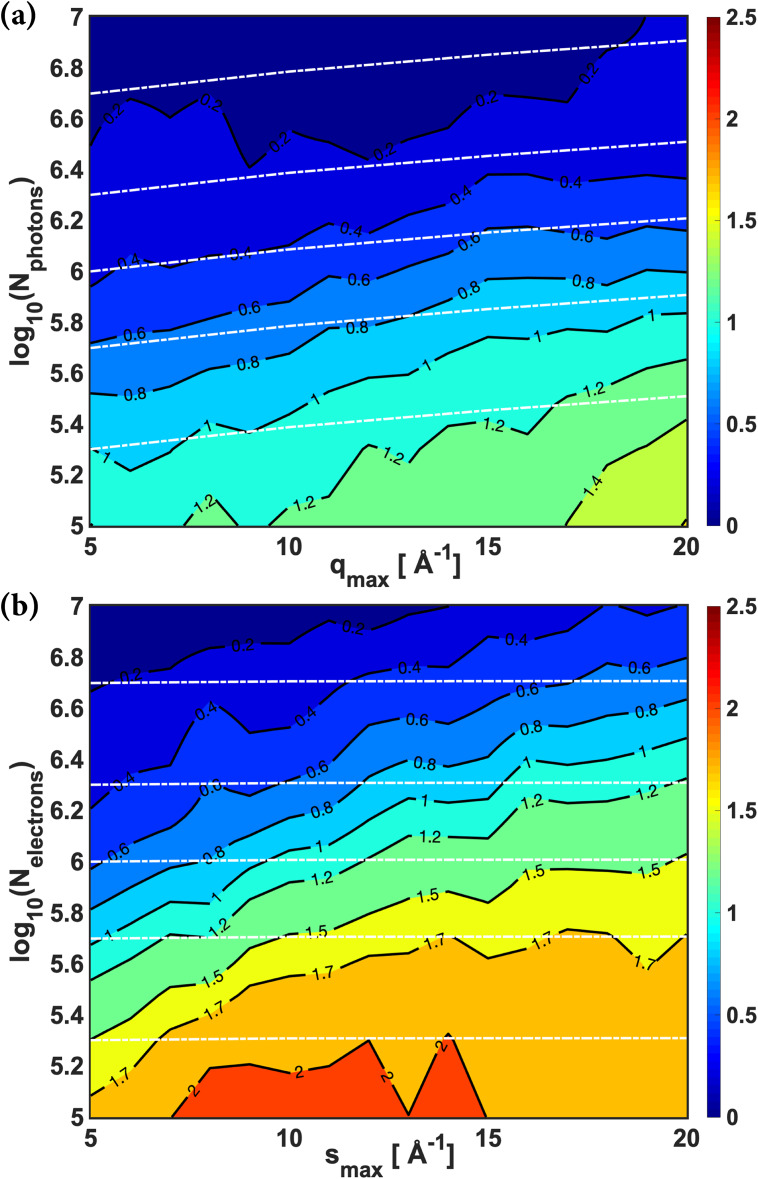
The accuracy of the structure determination, calculated as the average percent deviations as defined in Eq. (S1), as a function of the number of scattered probe particles (*N_photons_* and *N_electrons_*) and the momentum transfer *q* or *s* for x-ray scattering (a) and electron scattering (b), respectively. All data are calculated for a range of scattering vectors starting at 0.5 Å^−1^ and reaching to *s_max_* and *q_max_* as given on the abscissa. See text for details. Bluer colors imply a higher accuracy structure determination. The white dashed lines show the total counts of scattered probe particles necessary to counter the dilution by increasing the range of scattering vectors for x-ray and electron scattering.

We determine structures for scattering ranges from 0.5 Å^−1^ to *q_max_* or *s_max_* and vary the maximum values from 5 to 20 Å^−1^. For each range of scattering vectors, the structure determination is repeated for 10–50 simulations to minimize errors from the randomness of the scattering process simulations, resulting in scatter plots of the average percent deviations vs the number of scattered probe particles. This distribution is fitted with an exponential form, $f\left(x\right)=ae^{bx}+ce^{dx},$ and the limiting values are subtracted, resulting in a dependence of the average percent deviation on the number of scattered probe particles for each scattering range (Fig. 5). The limiting values of the two methods are subtracted as discussed above.

Figure 5 shows that for both x-ray and electron scattering, a larger number of detected signal counts always result in a more accurate structure determination, regardless of the range of scattering vectors. Surprisingly, to reach the same fidelity of structure determination, electron scattering requires some 3 times higher signal counts than x-ray scattering (see details in the supplementary material, Table S4). The most likely explanation is that the signal counts in electron scattering are very tightly concentrated at small scattering angles (Fig. 3). This results in very low noise for small *s* but high noise at larger *s* (Fig. 4). The structure determination requires a good signal at large scattering vectors but that is where the noise in electron scattering becomes large. This unevenness in the noise results in the need for a higher total count to get a structure determination of similar quality as in x-ray scattering. Thus, it is the rapid 1/s^4^ dependence of the Rutherford scattering that ultimately leads to larger required total signal counts.

It is a widely held belief that larger ranges of scattering vectors always give a better structure determination and that ranges as low as 5 Å^−1^ would not carry sufficient information content. Figure 5 dispels both those beliefs. As is apparent, even the smallest scattering ranges can result in high fidelity structure determinations.^87^ This is because the determination of the structure depends on both the scattering range and the noise. For the smallest scattering angles, the noise is very small in both x-ray and electron scattering. The benefit of the small noise outweighs the drawback for the limited range of scattering vectors so that an accurate structure determination is possible for small organic molecules without heavy atoms. This insight is fully consistent with the determination of excellent excited state structures performed in Ref. 29 with a *q* range only up to 4.5 Å^−1^.

Interestingly, increasing the range of scattering vectors can have a detrimental effect on the structure determination: to reach the same fidelity structure with a larger range requires more signal counts than with the lower range of scattering vectors. In part, this is because the same signal counts get spread over a larger range of scattering angles, thereby diluting the signal at small scattering angles. The white dashed lines overlaid in Fig. 5 illustrate the increase in total counts necessary to counter that dilution. For example, if there are 1 million probe particles when $q_{\text{max}}$ or $s_{\text{max}}$ equals 5 Å^−1^, it requires 1.62 million photons or 1.02 million electrons to compensate the dilution effect when $q_{\text{max}}$ or $s_{\text{max}}$ increases to 20 Å^−1^ for x-ray and electron scattering, respectively, and to have the same 1 million counts in the range up to 5 Å^−1^.

As is seen, especially in electron scattering where the dilution is small, the average percent deviation increases beyond that dilution effect. This is likely due to the addition of noise in high scattering ranges that override some of the information contained within the lower scattering range and thus contaminate the accuracy of structure determination. Therefore, even though increasing the range of momentum transfers can increase the structural information contained in the scattering pattern, the addition of noisy data at the high end worsens the overall performance of the structure determination. To fully take advantage of the high scattering ranges, it is important to obtain sufficient signals to achieve low noise at high scattering angles. This is particularly challenging for electron scattering.

It is then clear that the $\frac{1}{s^{4}}$ dependence of the electron scattering signal has an adverse effect on the fidelity of structure determination because of the uneven distribution of noise. Small ranges of scattering vectors are suitable for the purpose of determining excited state molecular structures, and in that range, x-rays have an advantage even when comparing equal total signals. This compounds the advantage of current XFEL technology in producing scattering patterns with high signal counts.

#### Sensitivity toward hydrogen atoms

It is widely known that neutron scattering is the method of choice for measuring hydrogen atoms and that both electron scattering and x-ray scattering are challenged in this regard. That said, it is interesting to compare the relative ability of electron and x-ray scattering for determining the positions of hydrogen atoms in molecules. Inspection of Fig. 2 suggests that beyond 5 Å^−1^, the x-ray scattering cross section for hydrogen atoms becomes very small. Yet, we saw that molecular structures are well determined by scattering experiments going to 5 Å^−1^ so that it may appear possible to measure hydrogen in x-ray experiments. At the same time, the electron scattering form factor for hydrogen atoms is small at low *s*, where electron scattering is strong, but rapidly approaches its terminal value of 1 beyond 5 Å^−1^. This leads one to expect a sensitivity of electron scattering to hydrogen atoms, but it would be at higher scattering angles that are challenging as discussed above.

**FIG. 6.**
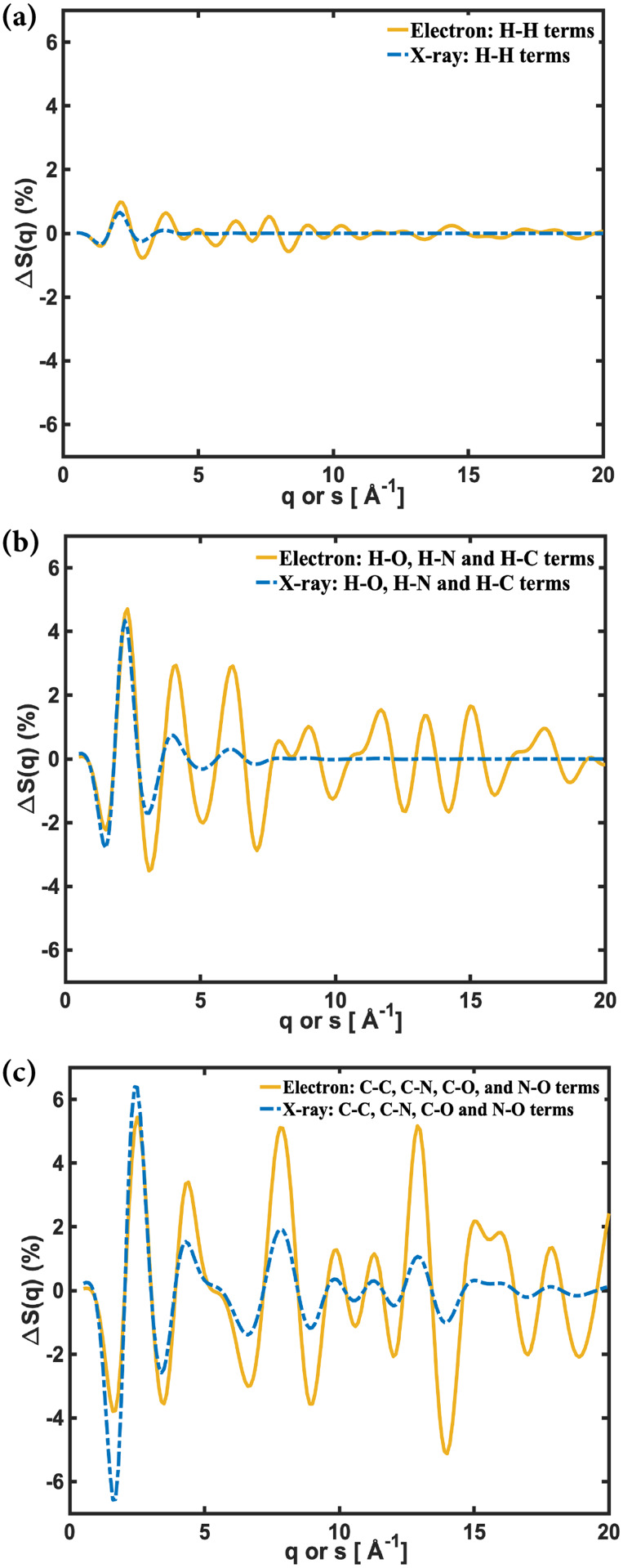
The contributions to the fractional difference scattering signals of Fig. 4 arising from (a) the H–H interatomic distances, (b) the H–O, H–N, and H–C interatomic distances, and (c) the skeleton atom interatomic distances.

The different shapes of the atomic form factors lead to slightly different molecular scattering signals, as it is already seen in Fig. 4. We can use the IAM simulations to trace the differences in the scattering signals to different groups of component atoms (Fig. 6). Starting with the carbon, nitrogen and oxygen atoms, Fig. 6(c), the two scattering experiments give the same peaks and valleys in the signals. However, the modulations are larger at higher scattering vectors for electron scattering than for x-ray scattering. This is because the fractional difference signals, calculated using Eq. (14), have *P_off_* in the denominator. For electron scattering, this is a steeply declining function, while for x-ray scattering, it levels off (Fig. 3). Thus, we see that the difference between the curves for these atoms arises from the scaling employed to analyze the data.

The effect of different hydrogen form factors becomes apparent in Figs. 6(a), the H-H terms, and Fig. 6(b), the H–X (X = O, N, C) terms. Since there are 11 H atoms in NMM, the absolute contribution of the terms containing H atoms (H–H, H–O, H–N, and H–C) below about 5 Å^−1^ is substantial and, in fact, comparable in magnitude to the skeleton interatomic distances. Since that range is sufficient for structure determination, this suggests that both experiments could be able to determine the positions of hydrogen atoms.

Figures 6(a) and 6(b) also show that the modulations in the electron scattering signal persist to high scattering ranges, while in x-ray scattering, they quickly decay, beyond what one would expect from the scaling by the *P_off_* term. This is the direct result of the decaying x-ray form factor (Fig. 2). Consequently, electron scattering should be the method of choice for detecting hydrogen atoms, provided that signals can be measured to high *s* with low noise.

**FIG. 7.**
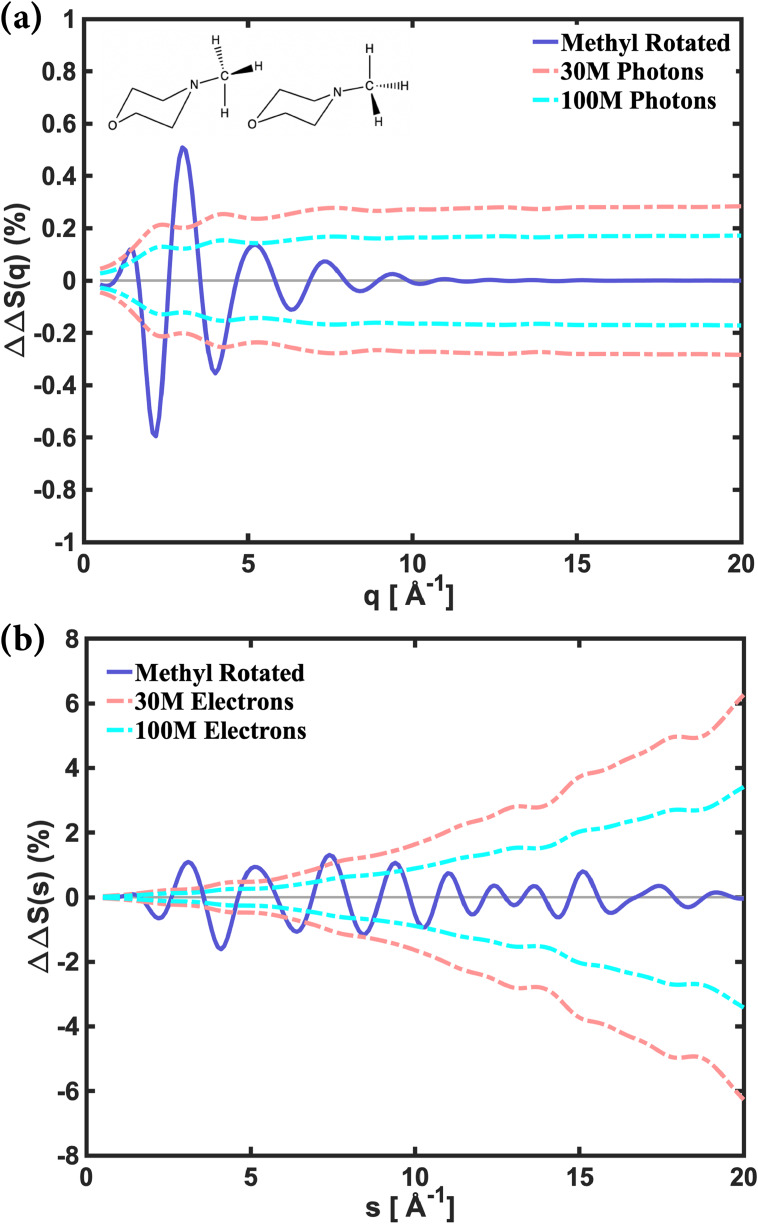
The difference between the fractional difference signals of two NMM conformers arising from different orientations of the methyl group, for (a) x-ray and (b) electron scattering. The dashed lines are RMS noise calculated from Eq. (15) within the 30M- and 100M-photon or electron simulations.

To further explore the possibility of measuring hydrogen atom locations, we simulated the NMM molecule in the excited state with two orientations of the methyl group while keeping all other atom positions fixed. Both conformers have a hydrogen atom in the plane of symmetry, but the dihedral angle H–C–N–O of one conformer is cis, while the other is trans. The difference in the scattering patterns between those two is small, with amplitudes of 0.5% and 1% for x-ray and electron scattering, respectively, compared to about 10% for the difference between ground and excited state structures (see Fig. 7).

It is apparent that the effect of the methyl rotation is significantly more dramatic in electron scattering, Fig. 7(b), than in x-ray scattering, Fig. 7(a): the modulation depth is higher, and the modulations extend to much larger scattering vectors. This inherently offers the opportunity to measure hydrogen atom locations very accurately with electron scattering. As Fig. 7 also shows, about 30M detected photons should allow x-ray scattering to determine the orientation of the methyl group. Electron scattering does likewise, but to really take advantage of the extended modulations, much higher electron signals would need to be accumulated.

## CONCLUSIONS

A comparative analysis of x-ray and electron scattering shows that while both methods measure the molecular form factors and thus have comparable information content, their inverse dependence on the form factors coupled with the different scaling of the Thomson and Rutherford scattering cross sections on the scattering vector gives rise to complementary measurements of molecular structures. On an absolute scale, the Thomson scattering is much weaker than the Rutherford scattering. However, with current technology, there are more than 10^7^ times as many photons in an x-ray pulse than there are electrons in an electron pulse. Consequently, the probability for a molecule to scatter a photon from an x-ray pulse is about 400 times larger than the probability to scatter a relativistic electron. Since these considerations are on a per-pulse basis, it is clear that to reach comparable quality signals, many more shots need to be acquired with electron scattering than with x-ray scattering. This provides a guideline for the design of experiments and to plan data acquisition times. We note that traditional electron diffraction experiments measuring ground state molecular structures^88–90^ use electron beam currents of about 2 *μ*A, which is about 6 orders of magnitudes more than current-technology pulsed electron beam currents. Not surprisingly, very high quality data to large scattering vectors can be acquired with those continuous electron beams.

We investigated the effect of shot noise and the range of momentum transfer vectors on the accuracy of excited state structure determination. Surprisingly, we find that with 10^6^ detected scattered particles, even a limited range of scattering vectors, up to 5 Å^−1^, can give excellent fidelity structures in both scattering experiments. Extending the range of scattering vectors while keeping the number of scattered particles constant does not improve the accuracy of the structure determination but burdens the analysis with noisy data. This is particularly so for electron scattering, where the signal rapidly decreases with *s* and the noise rapidly increases. The unevenness of the noise leads to a reduced accuracy of structure determination, with the consequence that MeV UED needs more than 3 times the signal count compared to x-ray scattering to reach the same structure fidelity. Combined with the higher x-ray signal per pulse, this implies that to reach equal quality structures, about 1200 times as many shots are required for an electron scattering experiment.

With sufficient quality datasets, both electron and x-ray scattering should be able to determine the position of hydrogen atoms. Because of the more favorable atomic form factor, electron scattering could, in principle, be superior to x-ray scattering for hydrogen atom detection but only if sufficiently low noise can be achieved.

Looking forward, the state-of-the art of both ultrafast pulsed XFEL and MeV electron sources is rapidly evolving. With the upgrade to LCLS-II, it will be possible to deliver x-rays with 1000 times higher brightness, albeit initially only with limited photon energies. Our result that low noise is more important than larger *q* ranges should make it an attractive proposition to measure excited state structures with LCLS-II.

Likewise, higher repetition rate MeV electron sources are on the horizon. While with current technology one has to resort to very long data acquisition times, the future MHz repetition rates would pack many more pulses into a given time span. This would greatly broaden the impact electron scattering can have on the measurement of excited state structures and dynamics.

The discovery that 5 Å^−1^ is sufficient for accurate structure determination for gas-phase small organic molecules has the enticing implication that the range of larger scattering vectors can be used to probe phenomena outside of the present model. Simulations by Kirrander and Weber have shown that wavepackets created on excited state surfaces are mirrored in scattering patterns as intricately detailed modulations.^91^ These modulations are most visible between 7 Å^−1^ and 20 Å^−1^. Thus, one can envision that the low *q* range would be more suitable to give the primary time-dependent molecular structure, while the higher *q* range would better image the shape of the traveling wavepacket. This separation will be most interesting to explore in future experiments with higher-brightness x-ray and electron sources.

## SUPPLEMENTARY MATERIAL

See the supplementary material for the nuclear structures, partial integrated scattering probabilities, details and convergence of structure determination method, and comparative structure determinations.

## Data Availability

The data that support the findings of this study are available within the article and its supplementary material.

## CODE AVAILABILITY

The code that supports the findings of this study is available from the corresponding author upon reasonable request.
